# A Novel Synthesized Sulfonamido-Based Gallate—JEZ-C as Potential Therapeutic Agents for Osteoarthritis

**DOI:** 10.1371/journal.pone.0125930

**Published:** 2015-06-24

**Authors:** Shixiu Wei, Zhenhui Lu, Yunfeng Zou, Xiao Lin, Cuiwu Lin, Buming Liu, Li Zheng, Jinmin Zhao

**Affiliations:** 1 The Medical and Scientific Research Center, Guangxi Medical University, Nanning, 530021, China; 2 Guangxi Key Laboratory of Regenerative Medicine, Guangxi Medical University, Nanning, 530021, China; 3 Guangxi Colleges and Universities Key Laboratory of Regenerative Medicine, Guangxi Medical University, Nanning, 530021, China; 4 Department of Toxicology, School of Public Health, Guangxi Medical University, Nanning, 530021, China; 5 School of Chemistry and Chemical Engineering, Guangxi University, Nanning, Guangxi, 530004, China; 6 Guangxi Key Laboratory of Traditional Chinese Medicine Quality Standards, Guangxi Institute of Traditional Medical and Pharmaceutical Sciences, Nanning, 530022, China; University of Umea, SWEDEN

## Abstract

Gallic acid (GA) and its derivatives are anti-inflammatory agents reported to have an effect on osteoarthritis (OA). However, GA has much weaker anti-oxidant effects and inferior bioactivity compared with its derivatives. We modified GA with the introduction of sulfonamide to synthesize a novel compound named JEZ-C and analyzed its anti-arthritis and chondro-protective effects. Comparison of JEZ-C with its sources i.e. GA and Sulfamethoxazole (SMZ) was also performed. Results showed that JEZ-C could effectively inhibit the IL-1-mediated induction of MMP-1 and MMP-13 and could induce the expression of TIMP-1, which demonstrated its ability to reduce the progression of OA. JEZ-C can also exert chondro-protective effects by promoting cell proliferation and maintaining the phenotype of articular chondrocytes, as evidenced by improved cell growth, enhanced synthesis of cartilage specific markers such as aggrecan, collagen II and Sox9. Meanwhile, expression of the collagen I gene was effectively downregulated, revealing the inhibition of chondrocytes dedifferentiation by JEZ-C. Hypertrophy that may lead to chondrocyte ossification was also undetectable in JEZ-C groups. The recommended dose of JEZ-C ranges from 6.25×10^-7^ μg/ml to 6.25×10^-5^ μg/ml, among which the most profound response was observed with 6.25×10^-6^ μg/ml. In contrast, its source products of GA and SMZ have a weak effect not only in the inhibition of OA but also in the bioactivity of chondrocytes, which indicated the significance of this modification. This study revealed JEZ-C as a promising novel agent in the treatment of chondral and osteochondral lesions.

## Introduction

Cartilage defects are usually characterized by a structural breakdown due to trauma or disease, which may lead to chronic disabilities. After injury, catabolic factors are activated, such as pro-inflammatory cytokines that can inhibit chondrogenic differentiation and induce a gradual self-destruction of cartilage finally resulting in secondary osteoarthritis (OA) [1]. Interleukin-1 (IL-1), a well-known monocyte/macrophage product, inhibits the synthesis of proteoglycans and collagen and enhances their degradation [2–3]. A previous study showed that IL-1 mediated marked downregulation of the matrix metalloproteinases (MMPs) and upregulation of tissue inhibitor of metalloproteinase-1 (TIMP-1) in chondrocytes [4], which combined may stimulate cartilage senescence and destruction in OA patients. The ideal therapeutic agent for OA would not only reduce joint inflammation but would also maintain normal cartilage function [5]. Gallic acid (GA) and its derivatives are a group of polyphenol compounds that have been known to have strong anti-oxidant [6] and anti-inflammatory [7–8] properties through the modulation of several important pharmacological and biochemical pathways. Gallic acid has been reported to induce apoptosis of rheumatoid arthritis (RA) fibroblast-like synoviocytes (FLS) by regulating the expression of apoptosis-related proteins and reducing the expression of pro-inflammatory mediators, such as pro-inflammatory cytokines, chemokines, COX-2 and MMP-9 [9]. Another investigation revealed that gallic acid attenuates proinflammatory and pro-oxidant effects [10]. In addition, the bioactivity of GA is compromised because it is much more hydrophilic than its esters, resulting in much weaker anti-oxidant effects than its esters in cell systems [6]. GA has also been reported to suppress cell proliferation[11]. Therefore, the introduction of certain lipophilic compounds onto GA may improve its bioactivity and pharmacological effects.

The sulfonamide family, possessing a broad spectrum of synthetic bacteriostatic antibiotics, has been commonly used in the last century in human and veterinary medicine for therapeutic and prophylactic purposes for their ability to easily penetrate through membranes and into body fluids and tissues [12]. Nuti E and et al reported that compounds contained several phenyl groups and a sulfonamide group were effective in blocking ex vivo cartilage degradation without side effects on cytotoxicity [13]. Recently, we reported a new series of derivatives of GA synthesized by coupling with sulfonamides including sulfathiazole sodium, sulfadimidine, sulfachloropyrazine sodium and sulfamonomethoxine sodium [14–17]. These compounds were found to be effective in promoting proliferation and maintaining phenotype of chondrocytes. However, their effects on OA has not been elucidated. Our pilot study showed that GA modified with sulfonamides may exhibit easy absorption properties that may promote its pharmacological activity.

In this study, we synthesized a sulfonamido-based gallate, 3,4,5-trihydroxy-N-[4-(thiazol-2-ylsulfamoyl)-phenyl]-benzamide (JEZ-C) and tested its effect on IL-1-stimulated chondrocytes and the restoration of chondrocytes. Comparison of JEZ-C with its substrates, GA and SMZ, was also performed. This study may be helpful in developing a new agent for the treatment of OA.

## Materials and Methods

### The synthesis of JEZ-C

Electrospray ionization mass spectrum (ESI-MS) was recorded on a Shimadzu LC-MS 2010A. ^1^H and ^13^C NMR spectra were obtained from a Bruker Advance III 300 at 400 and 125 MHz, respectively.

**Fig 1:**
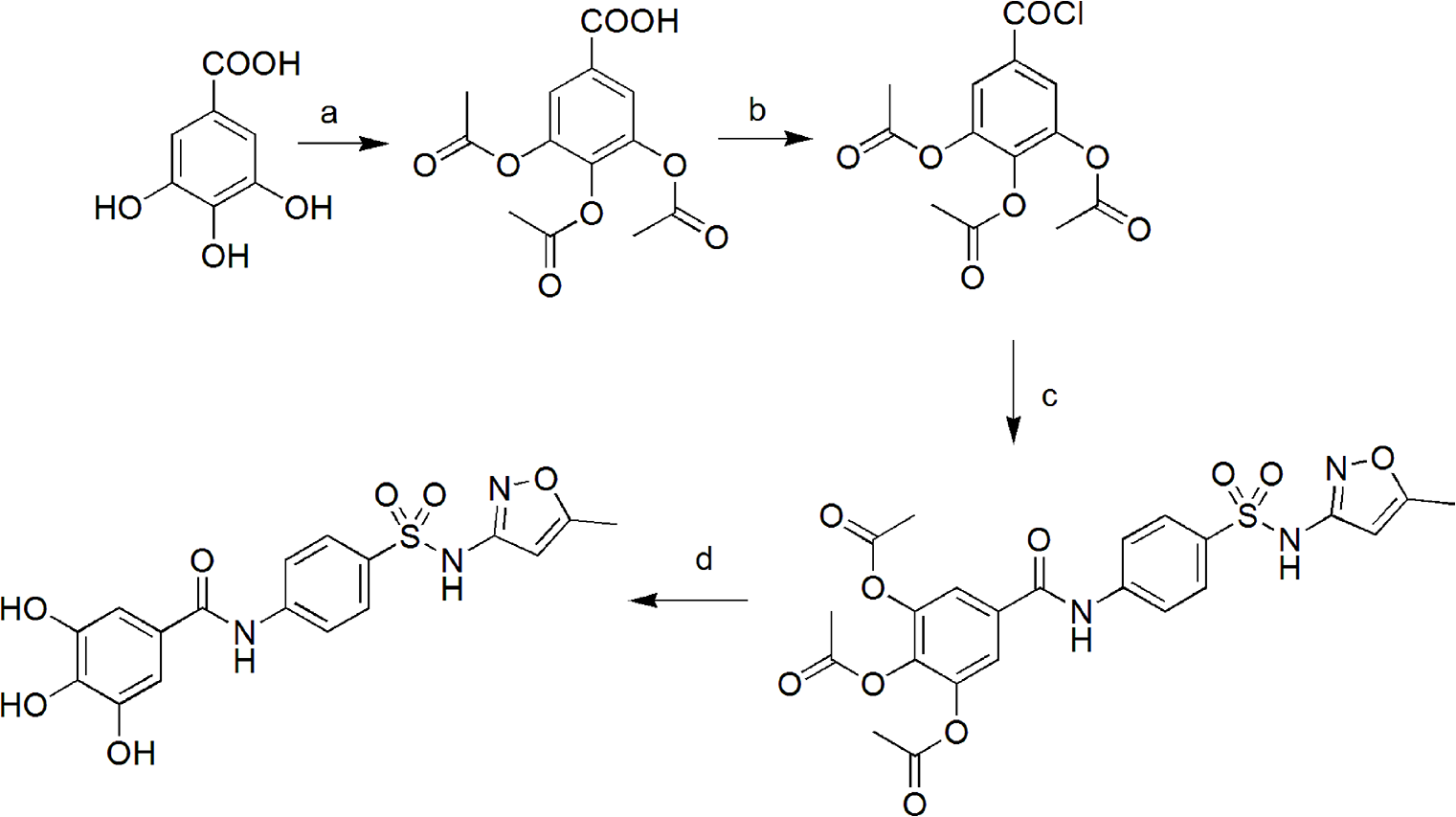
Reagents and conditions: (a) Acetyl oxide, oil bath, 120°C (b) SOCl_2_, oil bath, 80°C (c) Sulfamethoxazole, THF, Pyridine, ice bath (d) HCl, THF, 60°C.

3,4,5-Trihydroxy-N-[4-(thiazol-2-ylsulfamoyl)-phenyl]-benzamide (JEZ-C) was prepared from GA and Sulfamethoxazole with the same procedure in previous studies [18]. The synthetic route is presented in Fig 1 in detail. The purity of JEZ-C is greater than 95% by TLC (Thin Layer Chromatography, In three different development system only one spot appeared) and is 98% by HPLC (High Performance Liquid Chromatography) method.

### Articular chondrocytes culture

Articular chondrocytes were harvested from knee joint cartilage slices of 1-week-old rats by enzymatic digestion. In brief, cartilage slices from two rats which were injected Lethal of narcotics (pentobarbital sodium) and check the heart make sure death were dissociated enzymatically with 0.25% trypsin (Solarbio, China) for 30 min and then with 2 mg/ml collagenase type II (Gibco, USA) in alpha-modified Eagle’s medium (α-MEM, Gibco, USA) for 3 h. After centrifugation, the chondrocytes were resuspended. Cells were cultured with alpha-modified Eagle’s medium (α-MEM, Gibco, USA) containing 20% (v/v) fetal bovine serum (FBS) (Gibco, USA) and 1% (v/v) penicillin/streptomycin (Solarbio, China) in a 5% CO_2_ humidified incubator at 37°C with the culture medium replaced every other day after plating. Articular chondrocytes at passage 2 were used for further studies.

### Cytotoxicity assay

Cytotoxicity in chondrocytes was assessed by the 3-(4,5)-dimethylthiahiazo(-z-y1)-3,5-di-phenytetrazolium-romide (MTT, Gibco, USA) method. Articular chondrocytes were cultured with 200 μL of culture medium in 96-well microplates with preliminary treatment of various concentrations of JEZ-C, GA and SMZ for 2, 4 and 6 d. Twenty μl of 5 mg/ml MTT was added, and the plates were incubated in the dark at 37°C for 4 h. After removal of the MTT, cells were treated with 150 μl dimethyl sulfoxide (DMSO, Gibco, USA) to dissolve the formazan product. The absorbance was determined at 570 nm using an enzyme-labeled instrument (Thermo Fisher Scientific, UK).

As determined by MTT analysis, the concentrations of JEZ-C at 6.25×10^−7^, 6.25×10^−6^ and 6.25×10^−5^ μg/ml; of SMZ at 6.25×10^−6^, 6.25×10^−5^, and 6.25×10^−4^ μg/ml; and of GA at 0.078, 0.125, 0.156 μg/ml, among which a peak was presented, were chosen for further investigation.

### IL-1-induced chondrocytes

To investigate the effects of JEZ-C, GA and SMZ on IL-1 on the induction of chondrocytes, five groups were divided: (1) control group, chondrocytes without any treatment; (2) OA model group, chondrocytes treated with 10 ng/ml IL-1β (10 ng/ml, Gibco, USA); (3) JEZ-C treatment groups, chondrocytes pre-incubated with various concentrations of JEZ-C (6.25×10^−7^, 6.25×10^−6^, 6.25×10^−5^ μg/ml) for 1 h followed by stimulation with IL-1β for 24 h; (4) SMZ treatment groups, chondrocytes pre-incubated with various concentrations of SMZ (6.25×10^−6^, 6.25×10^−5^, 6.25×10^−4^ μg/ml) for 1 h followed by stimulation with IL-1β for 24 h; (5) GA treatment groups, chondrocytes pre-incubated with various concentrations of GA (0.078, 0.125, 0.156 μg/ml) for 1 h followed by stimulation with IL-1β for 24 h. The concentrations of JEZ-C, GA and SMZ were derived from the cytotoxicity assay.

### Cell proliferation analysis and biochemical assay

Chondrocytes were then continuously treated with JEZ-C, GA and SMZ for 2, 4, 6 d with the culture medium changed every 2 d. Cells treated for 2, 4 and 6 d were digested with proteinase K (Sigma, USA) for the following biochemical assay. Intracellular glycosaminoglycan (GAG) secretion was assayed with 1,9-dimethylmethylene blue (DMMB; Sigma, USA) dye, and the DNA content was quantified by Hoechst 33258 dye (Sigma, USA) assessment. The absorbance value of total intracellular DNA content in each sample was measured with a spectrofluorometer using Hoechst 33258 dye at 460 nm with calf thymus DNA as a standard. The total intracellular glycosaminoglycan (GAG) secretion was quantified spectrophotometrically at 525 nm with chondroitin sulfate (Sigma, USA) as a standard. Finally, the GAG content was normalized to the total DNA content of the chondrocytes.

### Morphological examination

Cells of the control, JEZ-C, GA and SMZ groups were removed from the incubator at 2, 4 or 6 d, and then were fixed in 95% alcohol for subsequent Hematoxylin-eosin (HE, JianCheng Biotech, China) staining. Cells were incubated with a nuclear dye for 3 min and then with a cytoplasmic dye for 5 s. Subsequently, the cells were rinsed by PBS, naturally dried and sealed with a neutral gum. Cells were then examined and photographed utilizing an inverted phase contrast microscope (Zeiss Corporation, Germany).

Another portion of the chondrocytes was used for the detection of actin filaments. In brief, cells were fixed with 4% paraformaldehyde (PFA, Sigma, USA) for 10 min at room temperature. After a rinse with PBS, cells were treated with 0.5% Triton X-100 (Sigma Aldrich, USA) for 5 min. Cells were treated with rhodamine phalloidin (Invitrogen, USA) for 30 min at room temperature in the dark to label the cellular matrix. After double-staining with Hoechst 33258 (Beyotime, USA) in the dark for 5 min, fluorescence was detected with a laser scanning confocal microscope (Nikon A1, Japan).

### Safranin O staining

Histology was performed to assess the synthesis of glycosaminoglycans (GAGs) using Safranin O staining. The cells, after being fixed by 95% alcohol for 30 min, were successively incubated with 0.1% Safranin O (Sigma, USA) for 10 min. Subsequently, the cells were rinsed with tap water and then dried naturally. Eventually, the cells were sealed with a neutral gum, observed and photographed by an inverted phase contrast microscope (Zeiss Corporation, Germany).

### Cell viability assay

Cell viability was determined with a live-dead viability assay kit (Invitrogen, USA). In brief, cells were quickly rinsed with PBS, and then, 1 μM calcein-AM and 1 μM PI were added to the cell cultures and incubated in the dark for 5 min at 37°C. After rinsing with PBS, the images were captured using a laser scanning confocal microscope (Nikon A1, Japan).

### Immunohistochemical staining

The secretion of collagen types I and II were performed immunohistochemically with an immunohistochemical staining kit (Bioss, China). To visualize protein, cells were fixed in 4% (w/v) paraformaldehyde and treated with Triton X-100. To exclude endogenous peroxidase activity, cells were incubated with 3% H_2_O_2_ for 10 min at room temperature. Cells were blocked with normal goat serum for 10 minutes at room temperature. After a 1:200 dilution of rat anti-rat antibody (collagen type I and II) was added, cells were then incubated with the second antibody and biotin labeled horseradish peroxidase. Subsequently, the antibody binding was visualized with a 3, 3’-diaminobenzidine tetrahydrochloride (DAB) kit (Boster, China) before brief counterstaining with hematoxylin. Eventually, cells were gradually dehydrated, sealed with a neutral gum, observed and photographed with an inverted phase contrast microscope (Zeiss Corporation, Germany).

### Gene expression

The genetic information was detected by qRT-PCR for type I, II and X collagen, aggrecan, Sox9, MMP-1, MMP-13 and TIMP-1(the primer sequences were showed in **Table 1**). Total intracellular RNA was extracted with an RNA isolation kit (Tiangen Biotechnology; Beijing, China) according to the manufacturer’s instructions. Approximately 300 ng of total RNA was used as a template and reverse transcribed into cDNA using a reverse transcription kit (Fermentas Company, USA). The qRT-PCR reactions were performed using a Quantitative PCR Detection System (Realplex 4, Eppendorf Corporation, USA) with a FastStart Universal SYBR Green Master (Mix, Roche company, Germany) under the condition of 10 min at 95°C, 15 s at 95°C and 1 min at 60°C. The melting curve data were collected to verify PCR specificity. Each gene was analyzed in triplicate to diminish operation errors. The relative gene expression levels were calculated using the 2^-ΔΔCt^ method using glyceraldehyde-3-phosphate dehydrogenase (GAPDH) as a control.

**Table 1:** Primers for RT-PCR performance.

Gene name	Forward primer	Reverse primer
Aggrecan	5’- CCGCTGGTCTGATGGACACT -3’	5’- AGGTGTTGGGGTCTGTGCAA -3’
Collagen II	5’- CTGGTCCTTCCGGCCCTAGA -3’	5’- GGATCGGGGCCCTTCTCTCT -3’
Sox9	5’-TCCAGCAAGAACAAGCCACA-3’	5’- CGAAGGGTCTCTTCTCGCTC -3’
Collagen I	5’- CATGAGCCGAAGCTAACCC -3’	5’- CTCCTATGACTTCTGCGTCTGG -3’
Collagen X	5’- TCTGCTGCTAGTGTCCTTGACG -3’	5’- GGAATGCCTTGTTCTCCTCTTACT -3’
MMP-1	5’-GGACTTGCTCACACATTCCCA -3’	5’- GAGTGAGTCCAAGGGAGTGG -3’
MMP-13	5’- GGATCCATGATGGCACTGCT -3’	5’- TGGCTTTTGCCAGTGTAGGT -3’
TIMP-1	5’- GCTTTCTGCAACTCGGACCT-3’	5’- TCTCCATGGCTGGGGTGTAG -3’
β-actin	5’-CCCATCTATGAGGGTTACGC -3’	5’-TTTAATGTCACGCACGATTTC -3’

### Statistical Analysis

Results were presented as the means±SD. Significant differences were determined using one way analysis of variance (ANOVA) followed by Dunnett’s post hoc test. The level of significance was set to P<0.05.

## Results

### Preparation of JEZ-C

The procedure of synthesis of GA and Sulfachloropyrazine sodium was shown in **Fig 1**, JEZ-C has the following properties: White powder, mp: 217–218°C, yield 63%, MS-ESI: 404.0[M-H]^-^, ^1^H-NMR (400 MHz, DMSO-d6) δ 11.33(s, 1H,-SO_2_-NH), 10.29(s, 1H,-CO-NH), 7.95–7.77 (m, 4H, 4×Ar-H), 6.94(s, 2H, 2×Ar-H), 6.13 (s, 1H, isoxazol-H), 2.28(s, 3H,-CH_3_). ^13^C-NMR (125 MHz, DMSO-d6) δ170.30, 166.09, 157.64, 145.60, 144.06, 137.37, 132.96, 127.83, 124.31, 119.79, 107.47, 95.43, 12.10

### Cytotoxicity assay

**Fig 2:**
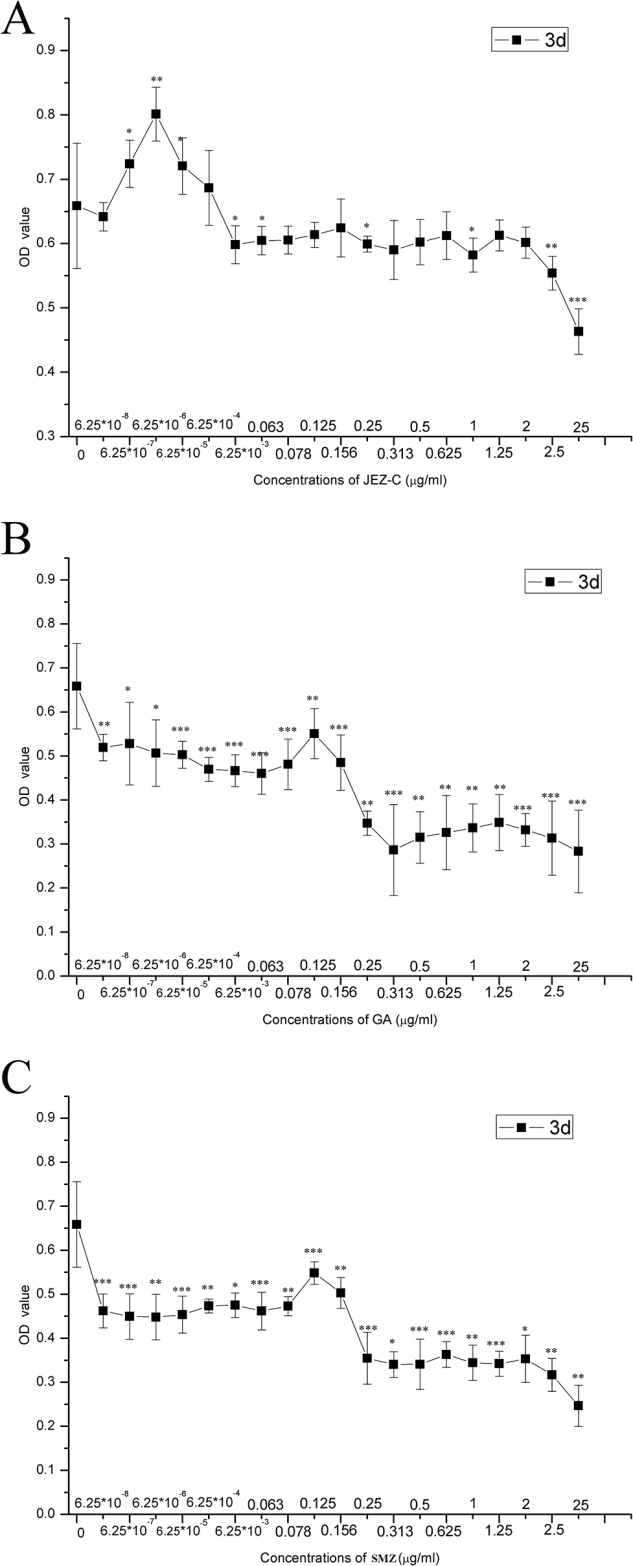
Cytotoxicity of JEZ-C(A), GA (B) and SMZ(C) on chondrocytes after 3 d (mean ± SD, n = 5). *p<0.05, **p<0.01, ***p<0.001.

This study examined the cytotoxicity of different drugs on articular chondrocytes by MTT assay. Articular chondrocytes were treated with JEZ-C, GA or SMZ in increasing concentrations (6.25×10^−8^ to 25 μg/ml). As shown in **Fig 2**, JEZ-C concentrations ranging from 6.25×10^−7^ to 6.25×10^−4^ μg/ml were comparable to the control and therefore nontoxic to cells. However, JEZ-C above 6.25×10^−3^ μg/ml exhibited an inhibitive effect on chondrocytes. Comparatively, SMZ and GA exhibited insignificant or inhibitive effects on chondrocyte growth ranging from 6.25×10^−8^ to 25 μg/ml (Fig 2). Hence, concentrations of JEZ-C over the range from 6.25×10^−7^ to 6.25×10^−5^ μg/ml, which can significantly improve the cell proliferation, were used in subsequent assays, whereas concentrations of SMZ and GA ranging from 6.25×10^−6^ to 6.25×10^−4^ μg/ml and 0.078 to 0.156 μg/ml, respectively, were utilized.

### Effects of JEZ-C, GA and SMZ on IL-1-induced chondrocytes

**Fig 3:**
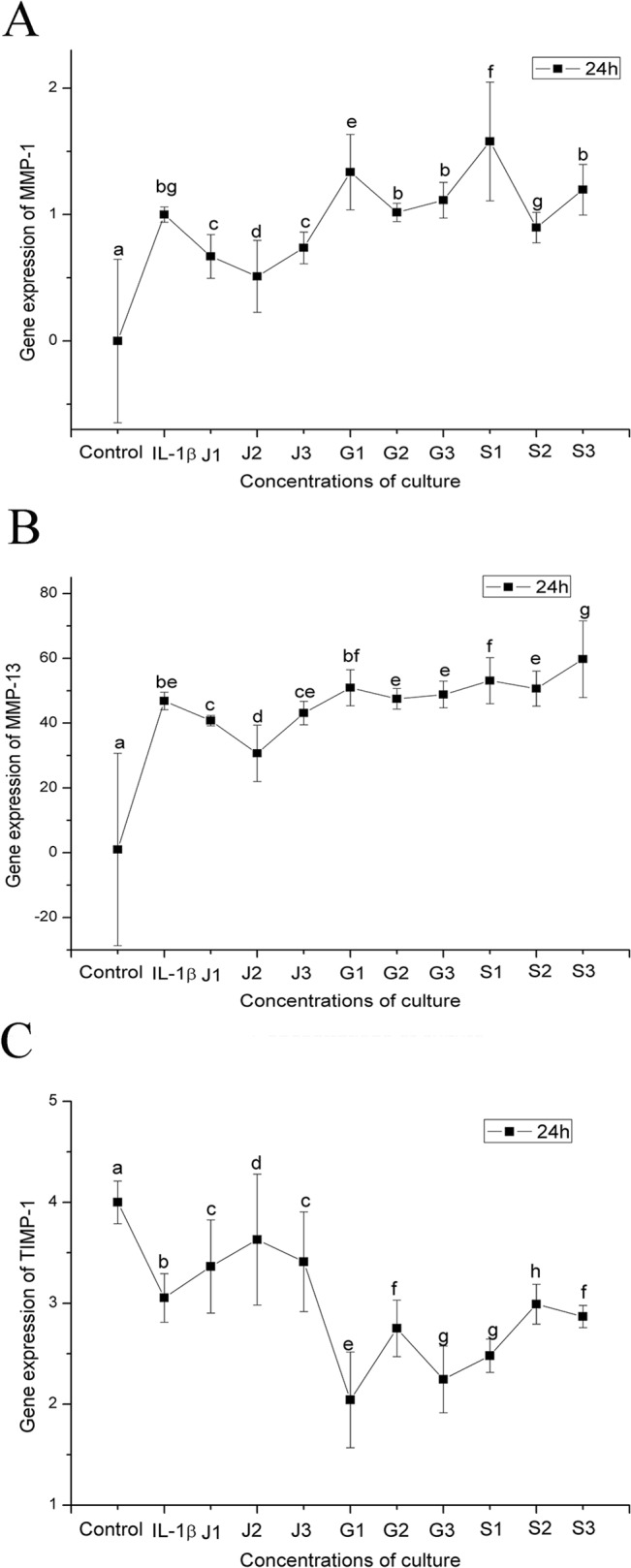
Quantitative comparison of ECM-related gene expression of MMP-1 (A), MMP-13 (B) and TIMP-1 (C) by qRT-PCR. The chondrocytes were cultured with different concentrations of JEZ-C, GA and SMZ: Control (without IL-1β), Model (with IL-1β), JEZ-C (J-1: 6.25×10^−7^ μg/ml; J-2: 6.25×10^−6^ μg/ml; J-3: 6.25×10^−5^ μg/ml), GA (G-1: 0.078 μg/ml; G-2: 0.125 μg/ml; G-3: 0.156μg/ml) and SMZ (S-1: 6.25×10^−6^ μg/ml; S-2: 6.25×10^−5^μg/ml; S-3: 6.25×10^−4^ μg/ml) on the induction by IL-1β for 6 days (n = 3 for each experiment). The gene expression levels in JEZ-C, GA and SMZ media relative to the control group were analysed by the 2^-ΔΔCT^ method using β-actin as the internal control. The data represent the mean±SD of three independent culture experiments. Bars with different letters are significantly different from each other at P﹤0.05.

**Fig 4:**
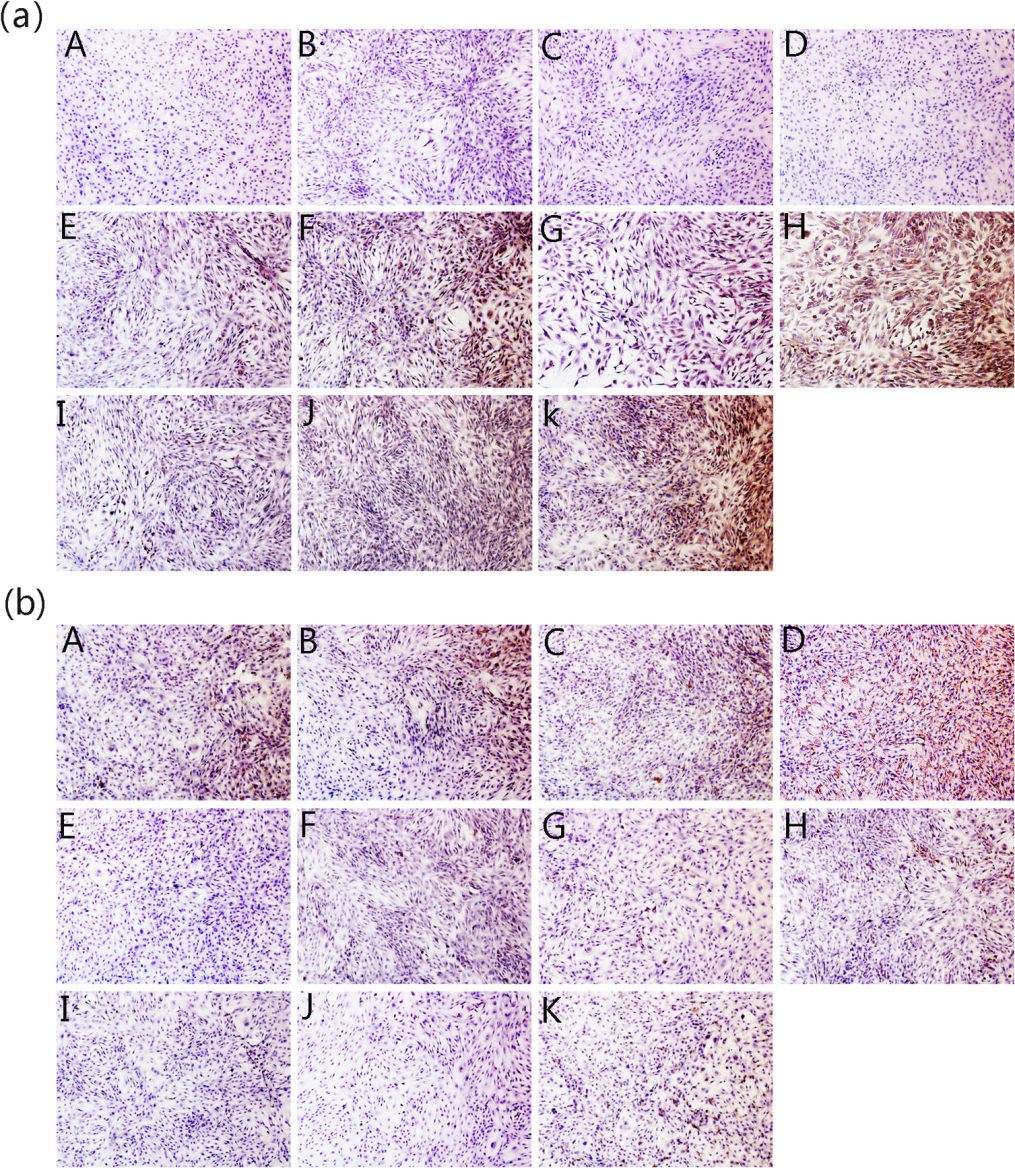
Immunohistochemical staining of MMP-1 (a) and TIMP-1 (b) of chondrocytes cultured *in vitro* with different concentrations of JEZ-C, GA and SMZ on the induction by IL-1β for 6 d: JEZ-C (A. 6.25×10^−7^ μg/ml; B. 6.25×10^−6^ μg/ml; C. 6.25×10^−5^ μg/ml); Control (D. without IL-1β); GA (E. 0.078 μg/ml; F. 0.125 μg/ml; G. 0.156 μg/ml); Model (H. with IL-1β); SMZ (I. 6.25×10^−6^ μg/ml; J. 6.25×10^−5^ μg/ml; K. 6.25×10^−4^ μg/ml), cell seeding density: 2×10^4^/ml (original magnification ×100). Scale bar = 200 μm.

Chondrocytes stimulated by IL-1β exhibited upregulation of MMP-1 and MMP-13 gene expression and downregulation of TIMP-1 expression. In contrast, JEZ-C inhibited the IL-1β-mediated induction of MMP-1 and MMP-13 gene expression and induced the expression of TIMP-1. However, SMZ and GA could not effectively prevent the induction of MMP-1 and MMP-13 gene expression or upregulate TIMP-1 expression (**Fig 3**). We next examined the effects of IL-1β, JEZ-C, GA and SMZ on protein secretion of MMP-1 and TIMP-1 in chondrocytes. The immunohistochemical results were in agreement with those of RT-PCR. These findings suggested that the IL-1β-mediated induction of MMPs and downregulation of TIMP-1 were effectively blocked by JEZ-C instead of SMZ or GA (**Fig 4**).

### Cell proliferation

**Fig 5:**
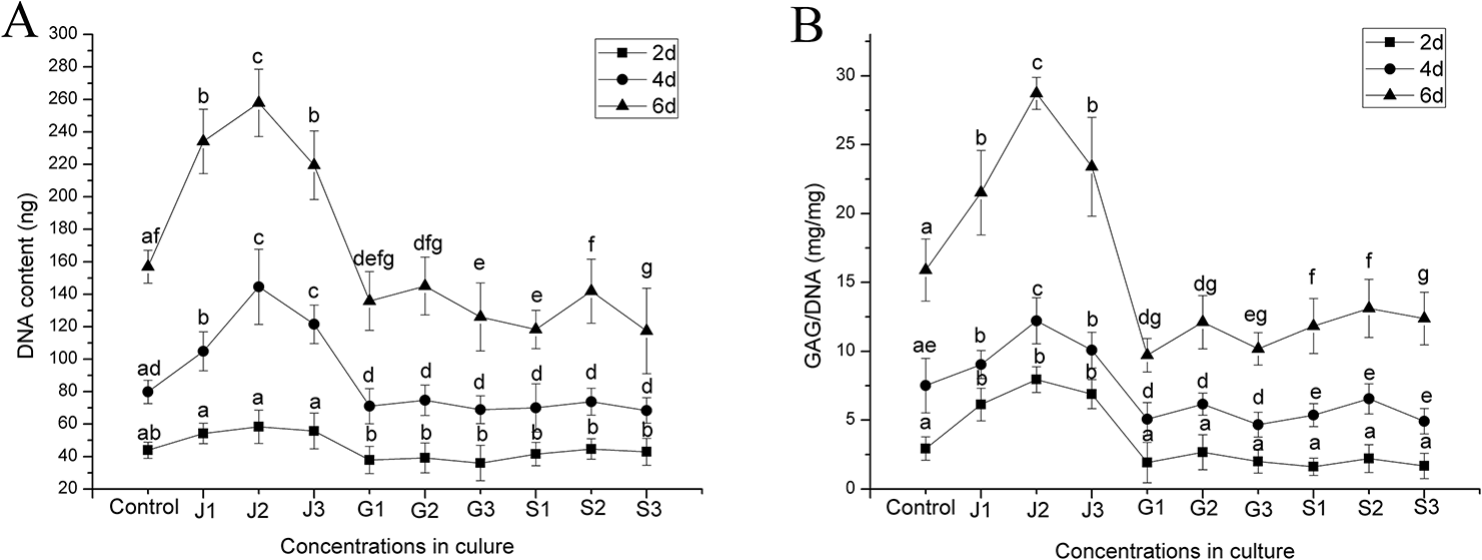
Quantification of cell proliferation (DNA) and matrix production (glycosaminoglycan (GAG)) of cells by biochemical assays: (A) the proliferation of chondrocytes cultured with different concentrations of JEZ-C, GA and SMZ: Control (K: 0 μg/ml), JEZ-C (J-1: 6.25×10^−7^ μg/ml; J-2: 6.25×10^−6^ μg/ml; J-3: 6.25×10^−5^ μg/ml), GA (G-1: 0.078 μg/ml; G-2: 0.125 μg/ml; G-3: 0.156μg/ml) and SDM (S-1: 6.25×10^−6^ μg/ml; S-2: 6.25×10^−5^μg/ml; S-3: 6.25×10^−4^ μg/ml) *in vitro* for 6 d; (B) GAG (mg) normalized to DNA (mg). Data from six independent experiments were evaluated and mean ±SD was showed, *P<0.05, **P<0.01, ***P<0.001.

In this study, the cell proliferation was analyzed by measurements of DNA content. Comparatively, cells cultured with JEZ-C grew faster than those in the control group (P﹤0.05), as shown by DNA values obviously higher than the control in the same culture period. Oppositely, cells treated with SMZ and GA grew slower than those in the control group (P﹤0.05). Furthermore, among all JEZ-C groups, the highest cell proliferation was achieved at a concentration of 6.25×10^−6^ μg/ml (**Fig 5A**).

### Secretion of GAGs

Results of intracellular GAG production treated by different concentrations of JEZ-C, GA and SMZ (**Fig 5B**) showed that GAGs gradually accumulated in all groups. Comparatively, GAG production in JEZ-C groups was significantly raised over that in the control at the same time point, whereas reduced tendency was obviously observed in SMZ and GA groups. Particularly, JEZ-C at a concentration of 6.25×10^−6^ μg/ml exhibited the strongest promotion of GAG synthesis among the three concentrations.

**Fig 6:**
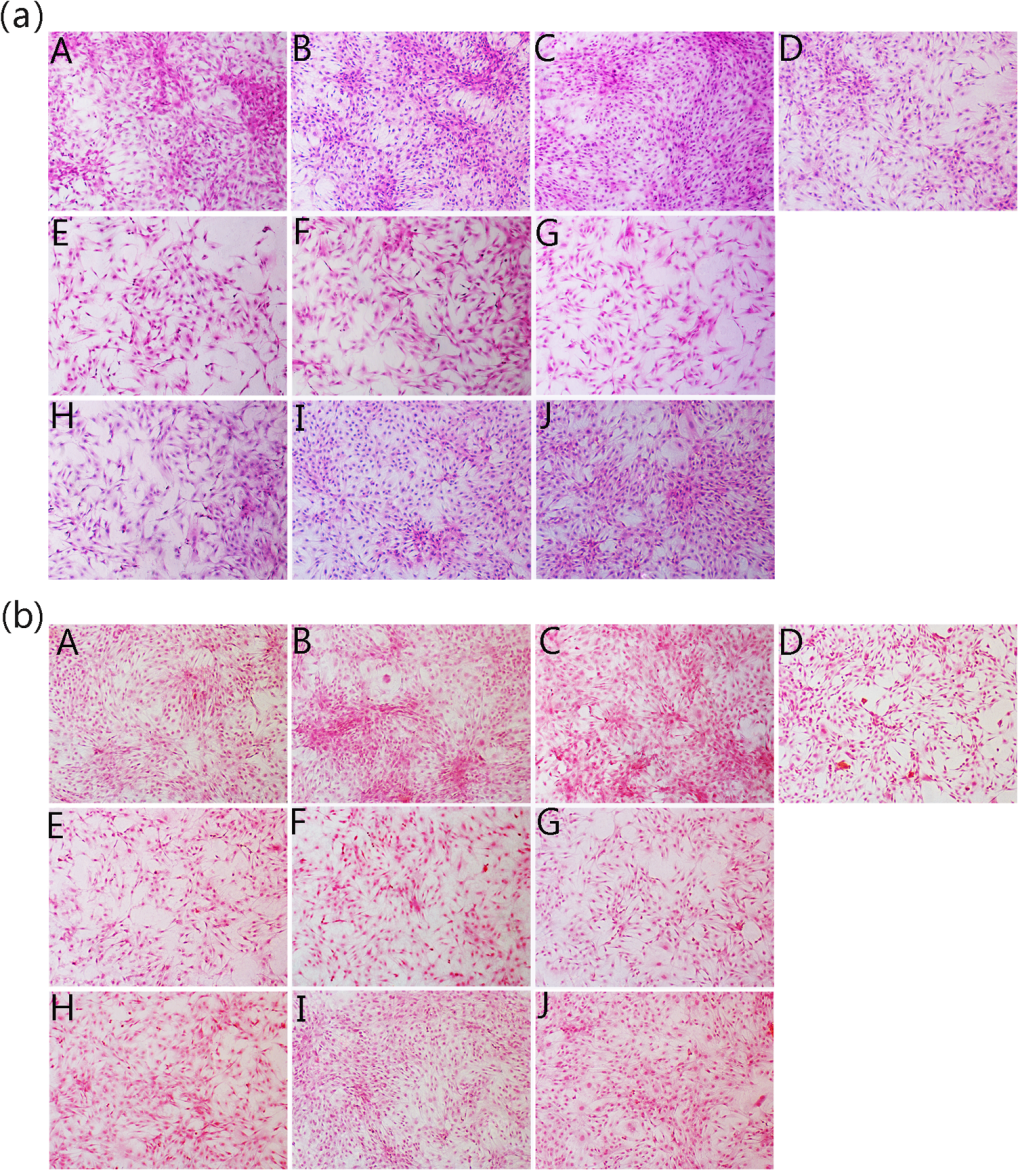
Hematoxylin-eosin staining (a) and Safranin O/fast green staining (b) images respectively showing the GAG production and the morphology of chondrocytes and cultured *in vitro* with different concentrations of JEZ-C, GA and SMZ for 6 d: JEZ-C (A. 6.25×10^−7^ μg/ml; B. 6.25×10^−6^ μg/ml; C. 6.25×10^−5^ μg/ml), Control (D. without IL-1β), GA (E. 0.078 μg/ml; F. 0.125 μg/ml; G. 0.156 μg/ml), SMZ (H. 6.25×10^−6^ μg/ml; I. 6.25×10^−5^ μg/ml; J. 6.25×10^−4^ μg/ml); cell seeding density: 2×10^4^/ml (original magnification ×100). Scale bar = 200 **μ**m.

The safranin O-positive stain (**Fig 6B**) in the JEZ-C groups indicated that GAGs were abundant and homogeneously distributed around the chondrocytes. Comparison of SMZ and GA groups with the control revealed indigent GAGs. The result of Safranin O staining conformed to GAG production by biochemical analysis (**Fig 5B**).

### Cell morphology

**Fig 7:**
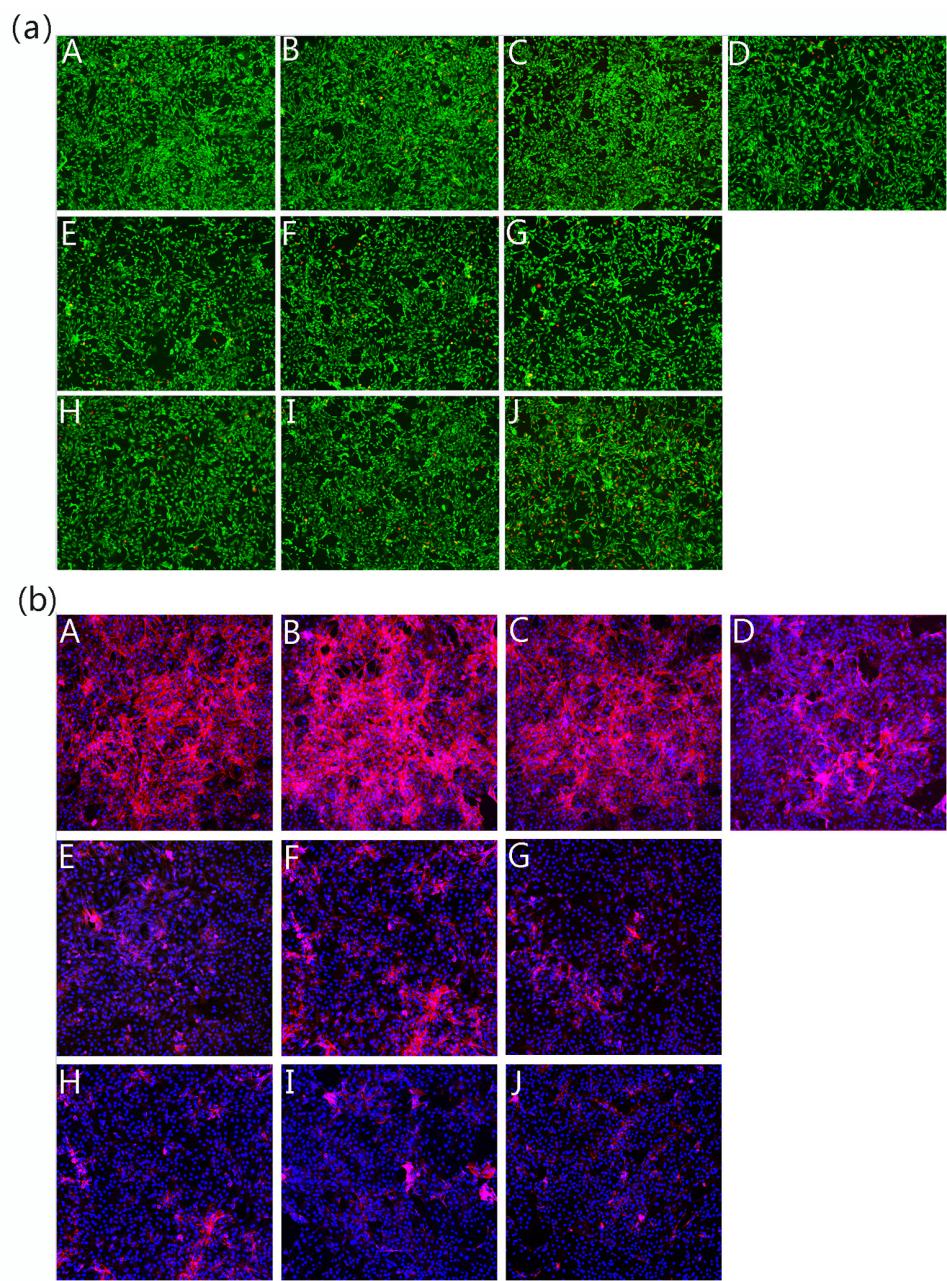
Confocal laser scanning microscopy images showing the viability (a) and distribution of the actin cytoskeleton (b) of chondrocytes cultured *in vitro* with different concentrations of JEZ-C, GA and SMZ for 6 d: JEZ-C (A. 6.25×10^−7^ μg/ml; B. 6.25×10^−6^ μg/ml; C. 6.25×10^−5^ μg/ml), Control (D. without IL-1β), GA (E. 0.078 μg/ml; F. 0.125 μg/ml; G. 0.156 μg/ml), SMZ (H. 6.25×10^−6^ μg/ml; I. 6.25×10^−5^ μg/ml; J. 6.25×10^−4^ μg/ml); cell seeding density: 2×10^4^/ml (original magnification ×100). Scale bar = 200 **μ**m.

Compared with the control, the chondrocytes in the presence of JEZ-C grew better and had a distinctive proliferation tendency that gradually increased with time (**Fig 6A**). In addition, at the concentration of 6.25×10^−6^ μg/ml, JEZ-C could better enhance the proliferation of chondrocytes over that of the other two concentrations. Nevertheless, these effects were not exhibited in the other two experimental groups treated with SMZ and GA. **Fig 7B** shows the actin filaments of chondrocytes by staining with rhodamine phalloidin/ Hoechst 33258, which was in agreement with the HE analysis. The cells in the JEZ-C treated groups grew in clumps with a densely distributed ECM. In the SMZ and GA groups, fewer cells and less ECM were present compared with the control.

### Cell viability assay

Viable cells and dead cells were determined using calcein-AM/PI staining (**Fig 7A**). The results demonstrated that JEZ-C exerts potent effects, whereas SMZ and GA demonstrated an inhibitory effect on chondrocytes survival under identical culture conditions. Calcein-AM/PI staining images displayed that survival in JEZ-C groups was higher than observed in the control. However, viable chondrocytes in the SMZ and GA groups was significantly less than that in the control. Consistent with the result of cell proliferation (**Fig 5A**), more viable cells were found than dead cells in the JEZ-C groups, implying that JEZ-C could better support cell growth than SMZ and GA. Among the JEZ-C groups, JEZ-C at a concentration of 6.25×10^−6^ μg/ml was superior to others, as evidenced by more viable cells.

### Secretion of type I and type II collagen

**Fig 8:**
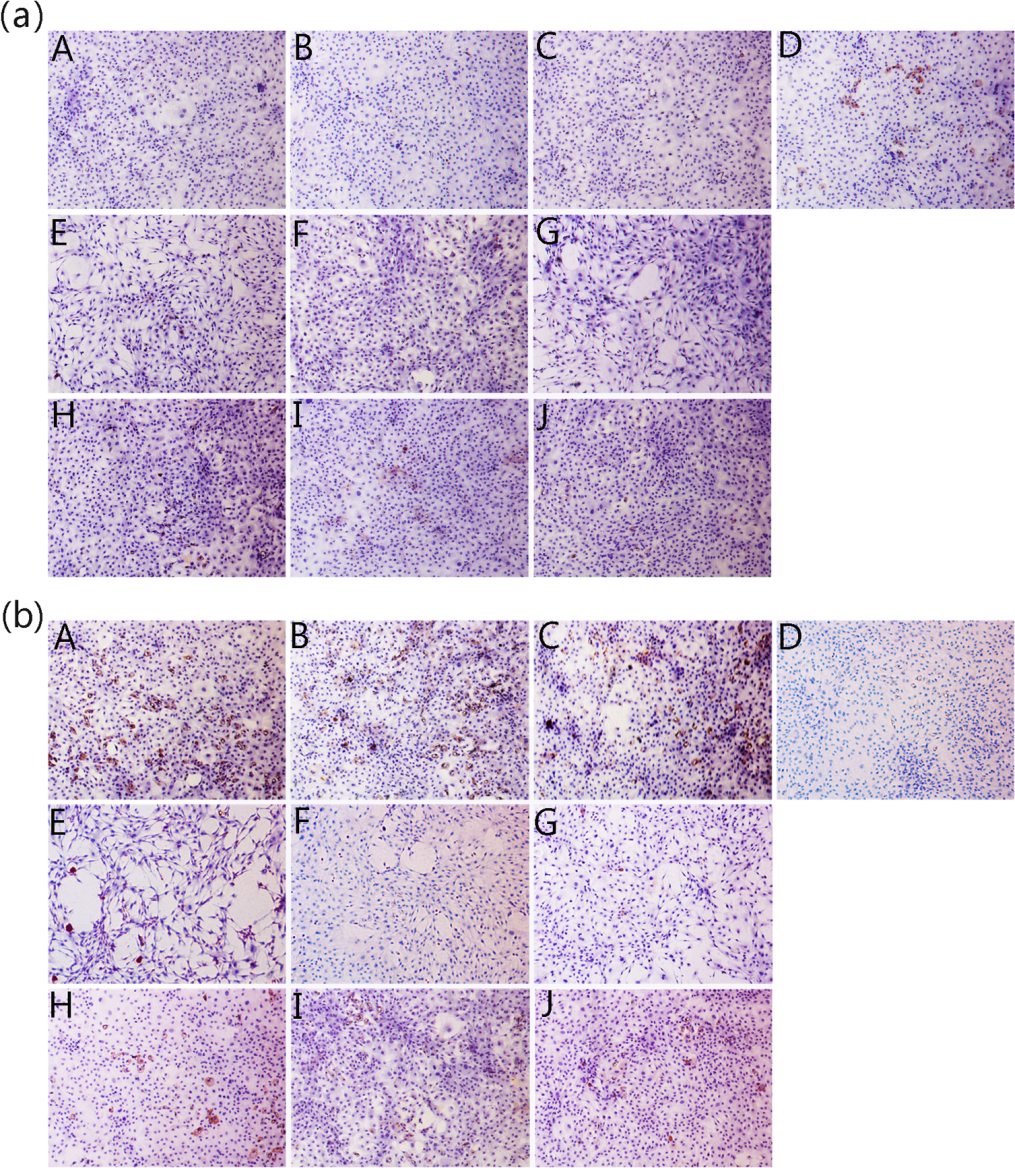
Immunohistochemical staining images revealed the presence of type I (a) and type II (b) collagen. Chondrocytes cultured *in vitro* with different concentrations of JEZ-C, GA and SMZ for 6 d: JEZ-C (A. 6.25×10^−7^ μg/ml; B. 6.25×10^−6^ μg/ml; C. 6.25×10^−5^ μg/ml), Control (D. without IL-1β), GA (E. 0.078 μg/ml; F. 0.125 μg/ml; G. 0.156 μg/ml), SMZ (H. 6.25×10^−6^ μg/ml; I. 6.25×10^−5^ μg/ml; J. 6.25×10^−4^ μg/ml); cell seeding density: 2×10^4^/ml (original magnification ×100). Scale bar = 200 μm.

Expression of type I and type II collagen in the cytoplasm at different levels with and without drug-treated culture media was shown in **Fig 8**. Strongly positive staining with large areas was evident for cartilage-specific type II collagen (**Fig 8B**) and only very sparse and light staining was seen for type I collagen (**Fig 8A**) in the JEZ-C groups after incubation for 2, 4 and 6 d, confirming the maintenance of the chondrocytic phenotype after treatment with JEZ-C. In addition, in the SMZ and GA groups, not only was collagen II staining weaker than the control but collagen I staining was also stronger than the control. These results indicated that JEZ-C may inhibit de-differentiation of chondrocytes cultured *in vitro* more effectively than SMZ and GA.

### Gene expression

**Fig 9:**
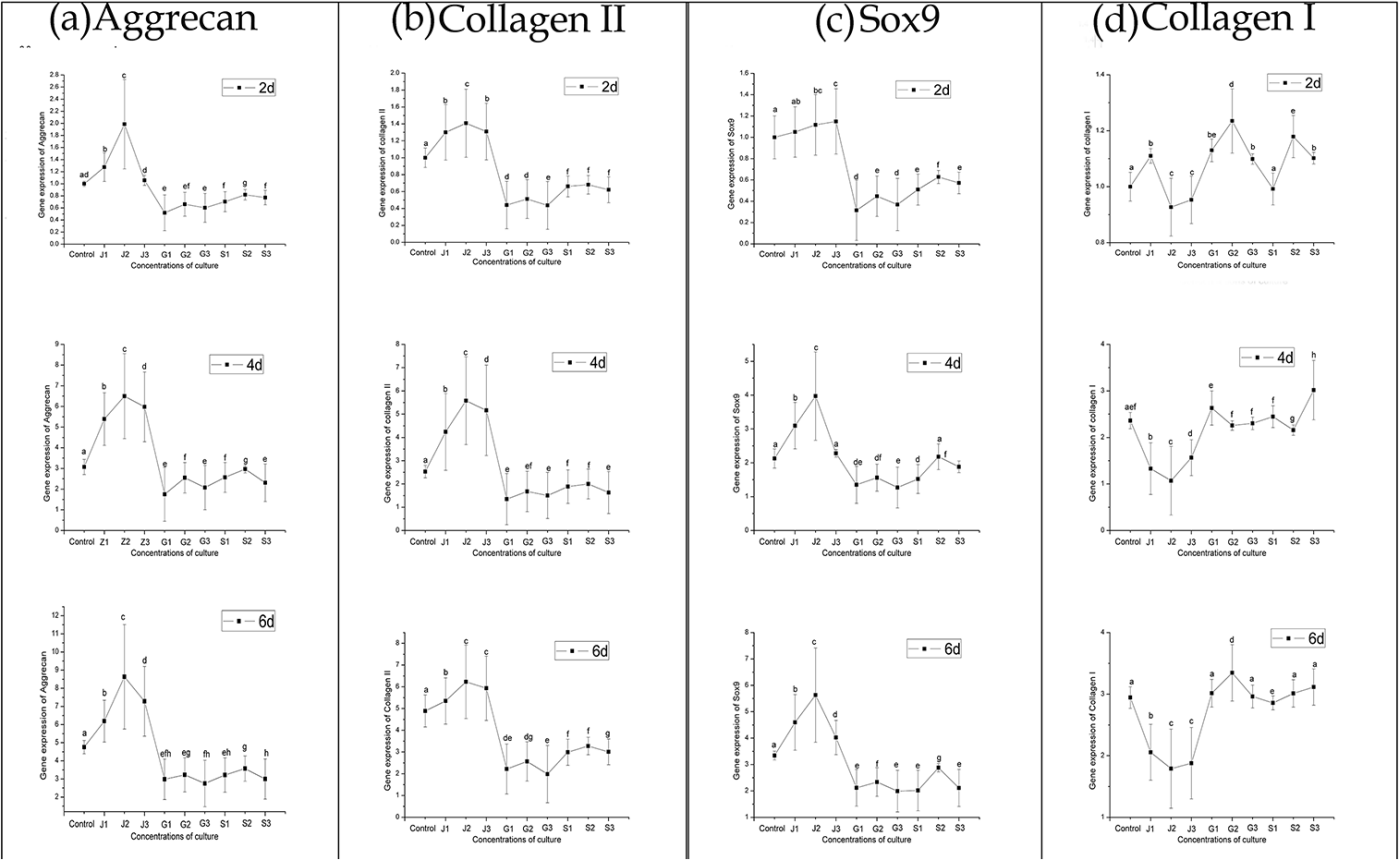
Quantitative comparison of ECM-related gene expression of aggrecan (a), collagen II (b), Sox9 (c) collagen I (d) and by qRT-PCR. The chondrocytes were cultured with different concentrations of JEZ-C, GA and SMZ: Control (K: 0 μg/ml), JEZ-C (J-1: 6.25×10^−7^ μg/ml; J-2: 6.25×10^−6^ μg/ml; J-3: 6.25×10^−5^ μg/ml), GA (G-1: 0.078 μg/ml; G-2: 0.125 μg/ml; G-3: 0.156μg/ml) and SDM (S-1: 6.25×10^−6^ μg/ml; S-2: 6.25×10^−5^μg/ml; S-3: 6.25×10^−4^ μg/ml) for 6 d (n = 3 for each experiment). The gene expression levels in JEZ-C, GA and SMZ media relative to the control group were analyzed by the 2^-ΔΔCT^ method using β-actin as the internal control. The data represent the means ± SD of three independent culture experiments. *p<0.05, **p<0.01, ***P<0.001.

As shown in **Fig 9**, cartilage specific gene expressions, such as aggrecan, collagen II and Sox9, were significantly boosted by JEZ-C at concentrations ranging from 6.25×10^−7^ to 6.25×10^−5^ μg/ml but were markedly reduced by SMZ and GA. Moreover, the highest collagen II, aggrecan and Sox9 expressions in the JEZ-C group were accompanied with the 6.25×10^−6^ μg/ml concentration. That JEZ-C up-regulated collagen II, aggrecan and Sox9 expressions suggested that JEZ-C either delayed or prevented the chondrocytes from dedifferentiating into a hypertrophic phenotype. Correspondingly, SMZ and GA may insignificantly influence the differentiation of chondrocytes. At the same time, collagen X expression was scarcely detectable in all groups, suggesting that cell hypertrophy was not prominent.

JEZ-C at different concentrations induced lower collagen I expression, whereas higher or similar collagen I expressions were surveyed in the SMZ and GA group when compared with the control group. Moreover, the levels of collagen I at a concentration of 6.25×10^−6^ μg/ml were lower than that of the other two concentrations in the JEZ-C groups. These results further hinted that JEZ-C, not SMZ and GA, could inhibit the dedifferentiation of chondrocytes.

## Discussion

GA was reported to have an effect on OA. However, GA has much weaker anti-oxidant effects than its esters and inferior bioactivity, which may be due to its hydrophilicity. It was hypothesized that synthetic compounds of gallates and sulfonamides may enhance its chondro-protective and pharmacological effects, Our previous studies demonstrated the chondro-protective effects of sulfonamido-based gallate [15–18]. But pharmacological effects, especially the effects on OA were still unknown. In this study, we synthesized JEZ-C and examined its effects on both chondro-protection and OA.

Many studies have demonstrated that IL-1 inhibits chondrocyte compensatory biosynthesis pathways, which can further compromise cartilage repair [19]. Chondrocytes stimulated with IL-1β in vitro have been exploited to imitate the microenvironment that occurs in osteoarthritis (OA) [20]. IL-1β is known to exert a key role in cartilage degradation through the induction of MMPs secreted by chondrocytes. Targeting MMPs is a promising approach to the treatment of OA because the MMPs, particularly MMP-1 and MMP-13, are interstitial collagenases that degrade type II collagen in the cartilage, which is a critical step in the progression of OA. In the present study, we utilized IL-1β to induce MMP gene expression and then assessed the effects of JEZ-C, GA and SMZ on MMP induction in rat articular chondrocytes. We observed that JEZ-C inhibited the IL-1β-mediated induction of MMP-1 and MMP-13 and induced the expression of TIMP-1, a metalloproteinase inhibitor, in rat articular chondrocytes (**Fig 3**). However, its substrates, including GA and SMZ, exhibit weaker effects. These results demonstrated that JEZ-C has the potential to be developed as potent anti-inflammatory agent in the treatment of OA.

However, the results indicated that JEZ-C could observably promote chondrocytes proliferation (**Fig 5A**). JEZ-C also markedly promoted GAGs deposition in cultured chondrocytes, which was shown with a biochemical assay (**Fig 5B**). Proteoglycans (PGs) are important components of extracellular matrices [21] For all PGs, glycosaminoglycans (GAGs) constitute a major component of their molecular mass; moreover, GAG and a large number of water molecules generate the expansion pressure and make the cartilage flexible, which plays an important role in maintaining cartilage load-bearing capacity [22]. Consistent with the increase in GAG production, JEZ-C could upregulate the gene expression of cartilage-specific aggrecan, collagen II and Sox9 (**Fig 9**). Chondrogenic transcription factor Sox9 played a major role in an increased level of chondrogenesis [23], in particular activating co-expression with collagen type II [24–26]. In addition, extensive gene therapy approaches using viral methods to over-express Sox9 resulted in marked improvements in the secretion of cartilaginous matrix by articular chondrocytes, bone marrow-derived stem cells and nucleus pulposus cells [27–29]. These data hinted that JEZ-C could facilitate chondrocytes proliferation and stimulate exuberant cartilage matrix secretion.

In addition, the expression of collagen type I, which marks dedifferentiation of chondrocytes, was effectively inhibited by JEZ-C. Dedifferentiation happens when the differentiated phenotype of chondrocytes, primarily composed of type II collagen and cartilage-specific proteoglycans, is bereaved and replaced by a complex collagen phenotype consisting of the vast majority of type I collagen and a low level of proteoglycan synthesis [30–32]. Furthermore, collagen type X, which is specifically associated with hypertrophic chondrocytes and precedes the onset of endochondral ossification [33], was nearly undetectable in JEZ-C groups, implying that the hypertrophy of chondrocytes would not be induced by JEZ-C. As a consequence, the decreasing collagen I expressions and the inconspicuous expressions of collagen X might suggest that JEZ-C may be preventing the dedifferentiation and hypertrophy of chondrocytes.

Because GA possesses inferior pharmacological effects and biological properties, modification of GA may be meaningful. Epigallocatechin-3-gallate (EGCG), the ester of epigallocatechin and gallic acid, was found to repress the degradation of human cartilage proteoglycan and type II collagen and selectively inhibit ADAMTS-1, ADAMTS-4 and ADAMTS-5 [34]. Another study revealed that EGCG ameliorates IL-1β-mediated suppression of TGF-β synthesis and enhances type II collagen and aggrecan core protein synthesis in human articular chondrocytes [35]. A study reported that sulfonamides could also inhibit cell wall synthesis [36]. However, another study showed that sulfonamides were slightly cytotoxic in human keratinocytes and rat hepatocytes [37]. Furthermore, recent studies indicated that sulfonamide-based gallates effectively inhibited IL-1β induced osteoarthritis [14] and exerted effects on cartilage growth [15–18]. In this study, JEZ-C, as a novel derivative of GA, can better support the chondrocyte growth and maintain their phenotype than its sources, as evidenced by increased cell proliferation, upregulated cartilage specific matrix compared with GA and SMZ. This implied that suitable modification of GA may lead to the improvement of its pharmacological effects.

Our results demonstrated that the concentration of JEZ-C with respect to enhancing chondrocytes proliferation ranged from 6.25×10^−7^ to 6.25×10^−4^ μg/ml (**Fig 2**). DNA production of rat articular chondrocytes was enhanced in a dose-dependent manner when chondrocytes were cultured in the medium containing JEZ-C at concentration of 6.25×10^−7^μg/ml to 6.25×10^−5^ μg/ml, and the 6.25×10^−6^ μg/ml group supported the strongest cell proliferation and stimulated the greatest matrix secretion.

In conclusion, sulfonamido-based gallate JEZ-C may relieve IL-1 destruction as a means to reduce the progression of OA. This novel compound also exhibits a chondro-protective effect by promoting cell proliferation and maintaining the phenotype of articular chondrocytes. In contrast, its source products of GA and SMZ have weak effects, not only in the inhibition of OA but also in the bioactivity of chondrocytes, which indicated the significance of this modification. JEZ-C as a novel agent is promising in the treatment of chondral and osteochondral lesions. Further studies are needed to elucidate the underlying mechanism of chondro-protective effect of JEZ-C.
