# Correction: A Novel Synthesized Sulfonamido-Based Gallate—JEZ-C as Potential Therapeutic Agents for Osteoarthritis

**DOI:** 10.1371/journal.pone.0222154

**Published:** 2019-08-29

**Authors:** Shixiu Wei, Zhenhui Lu, Yunfeng Zou, Xiao Lin, Cuiwu Lin, Buming Liu, Li Zheng, Jinmin Zhao

There are errors in Fig 7. The images appearing in panels (a) C, F and G, as well as (b) G and H are incorrect. The authors have provided a corrected version of Fig 7 here.

**Fig 7:**
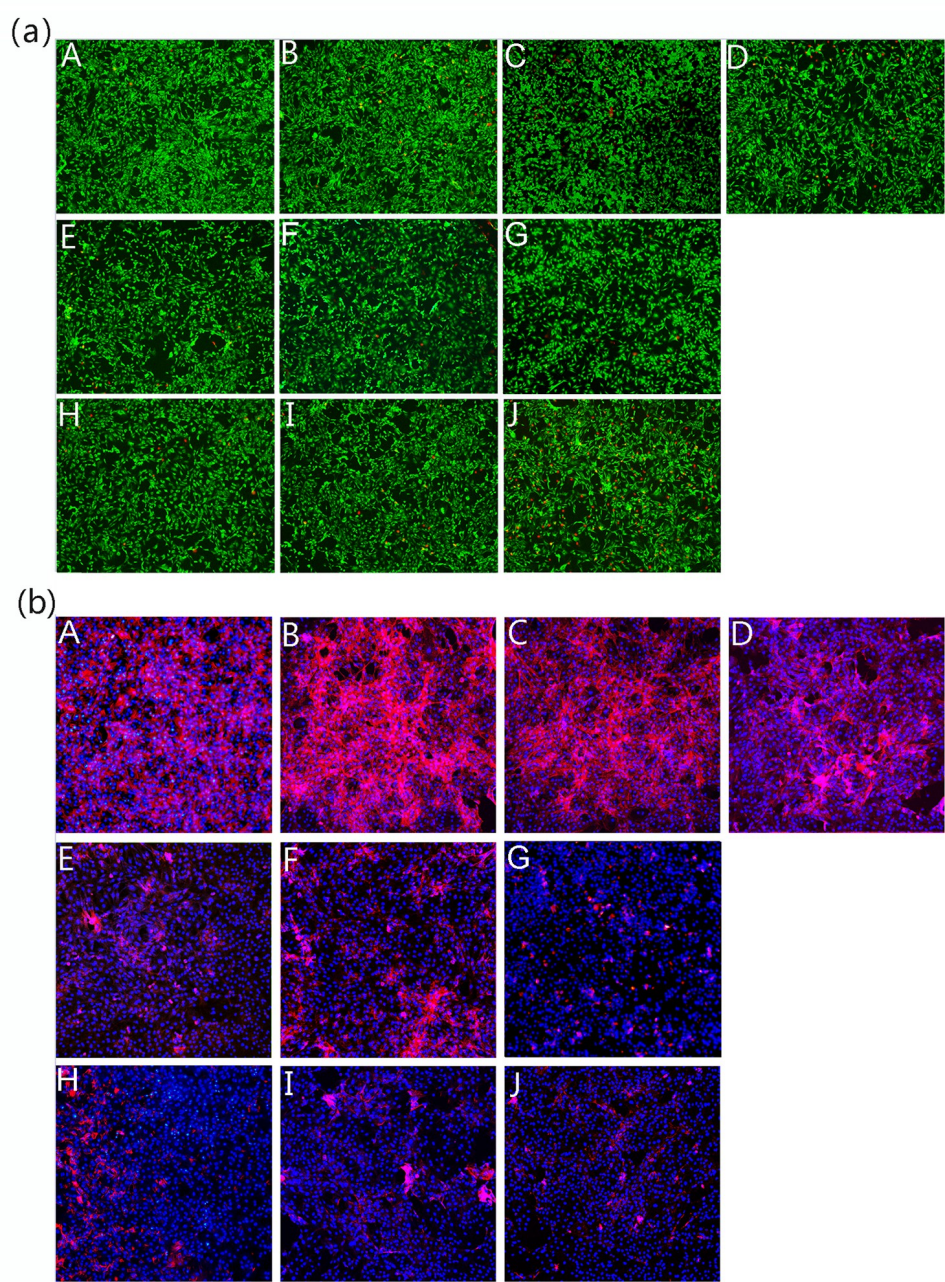
**Confocal laser scanning microscopy images showing the viability (a) and distribution of the actin cytoskeleton (b) of chondrocytes cultured *in vitro* with different concentrations of JEZ-C, GA and SMZ for 6 d: JEZ-C (A. 6.25×10**^**−7**^ **μg/ml; B. 6.25×10**^**−6**^ **μg/ml; C. 6.25×10**^**−5**^ **μg/ml), Control (D. without IL-1β), GA (E. 0.078 μg/ml; F. 0.125 μg/ml; G. 0.156 μg/ml), SMZ (H. 6.25×10**^**−6**^ **μg/ml; I. 6.25×10**^**−5**^ **μg/ml; J. 6.25×10**^**−4**^ **μg/ml); cell seeding density: 2×10**^**4**^**/ml (original magnification ×100).** Scale bar = 200 **μ**m.
